# Kidney transplantation is associated with reduced myocardial fibrosis. A cardiovascular magnetic resonance study with native T1 mapping

**DOI:** 10.1186/s12968-019-0531-x

**Published:** 2019-03-27

**Authors:** Mariana Moraes Contti, Maurício Fregonesi Barbosa, Alejandra del Carmen Villanueva Mauricio, Hong Si Nga, Mariana Farina Valiatti, Henrique Mochida Takase, Ariane Moyses Bravin, Luis Gustavo Modelli de Andrade

**Affiliations:** 1 Department of Internal Medicine, UNESP, Univ Estadual Paulista, Rubião Jr, s/n, Botucatu/SP, 18.618-970 Brazil; 2 Radiology Division, UNIFESP, Universidade Federal de São Paulo, São Paulo, Brazil; 3 Cardiology Division, UNESP, Univ Estadual Paulista, Botucatu, Brazil

**Keywords:** Cardiovascular magnetic resonance imaging, Fibrosis, Kidney transplantation, Native T1

## Abstract

**Background:**

The measurement of native T1 through cardiovascular magnetic resonance (CMR) is a noninvasive method of assessing myocardial fibrosis without gadolinium contrast. No studies so far have evaluated native T1 after renal transplantation. The primary aim of the current study is to assess changes in the myocardium native T1 6 months after renal transplantation.

**Methods:**

We prospectively evaluated 44 renal transplant patients with 3 T CMR exams: baseline at the beginning of transplantation and at 6 months after transplantation.

**Results:**

The native T1 time was measured in the midventricular septum and decreased significantly from 1331 ± 52 ms at the baseline to 1298 ± 42 ms 6 months after transplantation (*p* = 0.001). The patients were split into two groups through a two-step cluster algorithm: In cluster-1 (*n* = 30) the left ventricular (LV) mass index and the prevalence of diabetes were lower. In cluster-2 (*n* = 14) the LV mass index and diabetes prevalence were higher. Decrease in native T1 values was significant only in the patients in cluster-1 (*p* = 0.001).

**Conclusions:**

The native myocardial T1 time decreased significantly 6 months after renal transplant, which may be associated with the regression of the reactive fibrosis. The patients with greater baseline LV mass index and the diabetic group did not reach a significant decrease in T1.

**Electronic supplementary material:**

The online version of this article (10.1186/s12968-019-0531-x) contains supplementary material, which is available to authorized users.

## Background

Patients with chronic kidney disease (CKD) are at increased risk for cardiovascular disease (CVD) [1, 2]. Renal transplantation is associated with a reduction in cardiovascular mortality when compared to patients who remain on the transplant waiting list [3]. It is likely that the improvement in cardiac contractility is due to the regression of intermyocardiocytic fibrosis after transplantation [4]. Myocardial fibrosis affects more than 90% of patients with CKD [4] and leads to the accumulation of extracellular matrix (ECM) proteins in the cardiac interstice, which in turn results in increased left ventricle (LV) stiffness. This contributes to reduced diastolic filling, with the development of arrhythmias, heart failure and sudden cardiac death [5–8]. Hence, the extent and severity of myocardial fibrosis are important predictors of death among patients with CKD [4].

Three types of myocardial fibrosis have been reported: reactive interstitial fibrosis, infiltrative interstitial fibrosis and replacement fibrosis [9, 10]. Fibrosis may regress in the reactive type, whilst in the replacement one there is loss of myocytes due to necrosis and/or apoptosis [11–13].

Myocardial fibrosis is confirmed with endomyocardial biopsy, but it is a procedure with high morbidity [14, 15]. Imaging techniques that allow the detection of myocardial fibrosis are noninvasive methods that may improve tissue characterization and stratification of cardiovascular risk. On that account, cardiovascular magnetic resonance (CMR) imaging has been used to quantify myocardial fibrosis and replace endomyocardial biopsy. The measurement of extracellular volume fraction (ECV) by CMR is an efficient method to detect and quantify myocardial fibrosis [16, 17]. Although promising, ECV measurement uses gadolinium contrast, which is contraindicated in patients with a glomerular filtration rate lower than 30 mL/min due to the risk of nephrogenic systemic fibrosis [18].

As an alternative, myocardium native T1 mapping is a resonance method that evaluates myocardial and quantify the fibrosis without the use of gadolinium contrast [19, 20]. The native T1 strong correlates with histological myocardial fibrosis in patients with aortic stenosis [21]. In 2018, a meta-analysis established the reference values of native T1 for nonischemic cardiomyopathies and populations with increased cardiovascular risk [22]. Different types of tissue will have their own normal ranges of values, and a significant departure from the normal range is thought to differentiate between normal and abnormal myocardium.

It has already been demonstrated that in the hemodialysis population, native T1 values are higher in comparison with healthy subjects [20]. However, the values of native T1 after renal transplantation are not yet known.

The primary aim of this study was to evaluate the changing of septal native T1 between baseline and 6 months after renal transplantation.

## Patients and methods

This prospective single-center cohort study was performed in a tertiary medical center (Botucatu Medical School - UNESP, Univ Estadual Paulista). A total of 44 patients underwent two CMR examinations. The first exam (baseline) was performed between the 1st and the 10th postoperative days. The second one was performed 6 months after renal transplantation. Changes in the native T1 were evaluated by comparing the first and second exams of the same patient. The protocol was reviewed and approved by the institutional review board (CAAE: 40598414.9.0000.5411). Written informed consent was obtained from the subjects before participation.

### Inclusion and exclusion criteria

We included consecutive patients over 18 years of age who received a renal transplant from a living or deceased donor. We excluded patients with a contraindication to CMR (e.g., pacemaker, metal prosthesis, cochlear implant, cerebral aneurysm clip, tattooing, claustrophobia, hemodynamic instability) and inability to perform breath hold.

### CMR acquisition

All examinations were performed on the same 3 T CMR system (Magnetom Verio, Siemens AG, Healthineers, Erlangen, Germany) with a phased array chest coil, according to study protocol. A cardiac cine balanced steady-state free precession (bSSFP) sequence was acquired using retrospective cardiac gating. Typically, 25 phases were acquired in 2-, 3-, and 4-chamber long axis views and a stack of short axis views. Scan parameters: field of view (FOV) 37-cm, repetition time 43.54 ms, echo time 1.38 ms, flip angle 50°, slice thickness 6 mm, in-plane image resolution 1.6 × 1.6 mm. Quantitative T1 mapping was performed with a Modified Look-Locker Inversion-Recovery (MOLLI) sequence in mid-cavity short axis slice, without gadolinium (Native T1) following the recommendations of the European Society of Cardiology consensus [23]. Scan parameters: FOV 36-cm, repetition time 316.09 ms, echo time 1.12 ms, flip angle 35°, slice thickness 8 mm, in-plane image resolution 2.1 × 1.4 mm, acquisition in late diastole on every other heartbeat, minimal inversion time 120 ms; increment 80 ms. The T1 mapping scheme included 5 acquisitions after the first inversion pulse, followed by a 3-heartbeat pause and a second inversion pulse followed by 3 acquisitions [5(3)3 scheme].

### CMR analysis

**Fig. 1:**
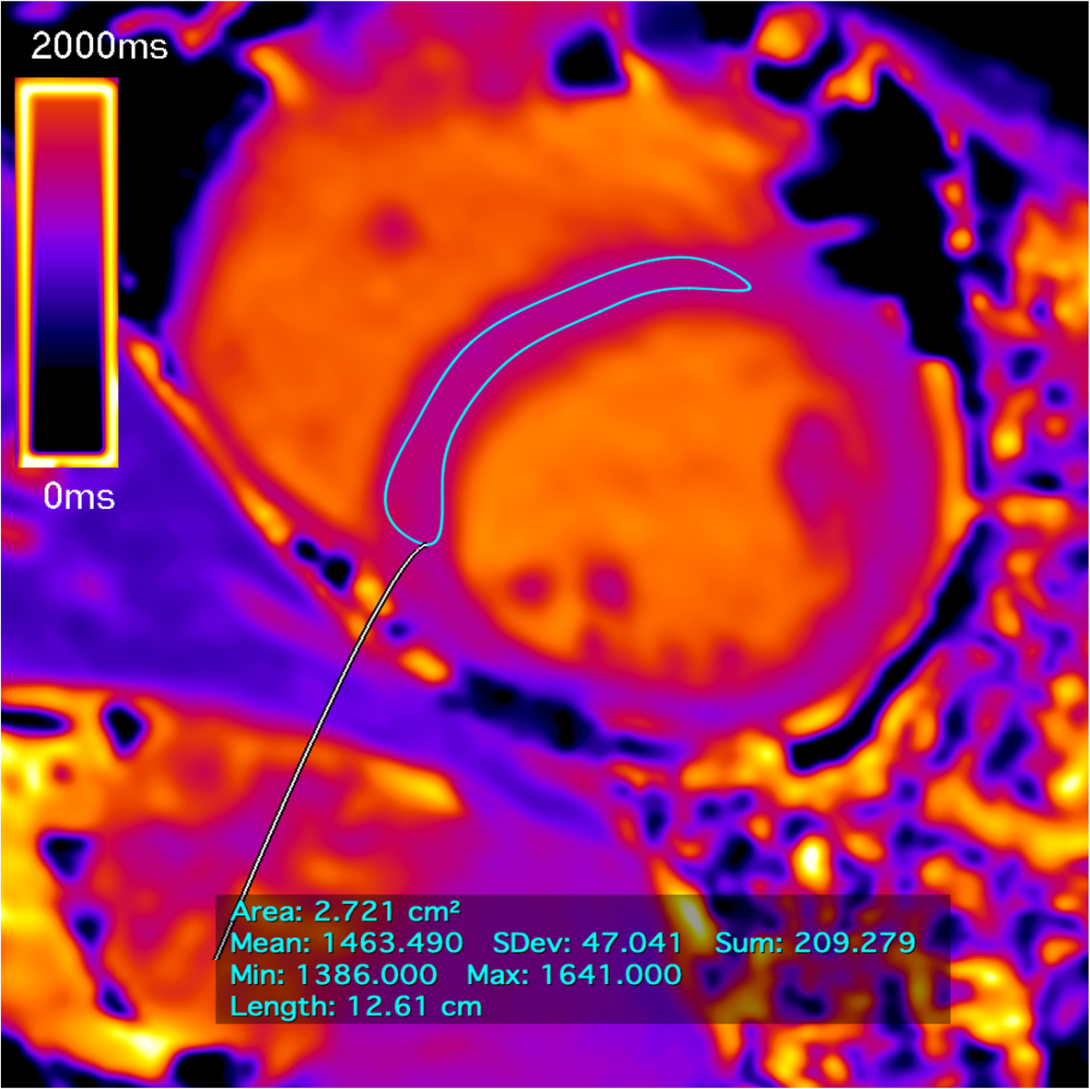
Example of T1 septal mapping. The thin line denotes the manually contoured region of interest (ROI) in the midventricular septum. The box shows the measures of T1 value by automatic computer average of the pixels. The color map scale represents the values in milliseconds (blue corresponds to smaller values and yellow to higher values). Data acquired with the modified look-locker inversion recovery (MOLLI) T1 mapping sequence at a field strength of 3 T

Biventricular ejection fraction, volumes and LV mass were calculated from the short-axis cine images with post-processing using Argus software (Siemens, Healthineers). Endocardial contours were drawn at both end-diastole and end-systole. LV epicardial contours were drawn only at end-diastole to calculate LV mass. This technique was repeated for each short-axis slice and the software calculated the volumes, mass and ejection fraction using the Simpson method. T1 maps were automatically generated on the CMR scanner. A region of interest (ROI) was then drawn in the septal myocardium to obtain T1 value (Fig. 1). The native T1 measurements using midventricular septum approach are recommended as the standardized due to independence of geometrical alterations of cardiac chamber and wall thickness [24]. All the patients underwent the same imaging protocol, analyzed by a single blinded observer, using the same technique of post-processing.

### Baseline characteristics and other clinical and laboratory parameters

Donor and recipient characteristics and immunosuppressive therapy were evaluated at baseline. Biochemical and renal function parameters and the use of non-immunosuppressive drugs between baseline and 6 months were evaluated. We also evaluated systolic and diastolic blood pressure and heart rate in the two time periods.

### Statistical analysis

#### Sample size calculation

A pilot study was conducted with 11 patients, in which a mean difference in native T1 of 50 ± 52 ms was found. Considering the alpha error of 0.05 and the beta of 0.8, for this study we calculated a number of 38 patients.

#### Descriptive statistics and comparisons between groups

Categorical variables were expressed in numbers and percentage and compared between the two moments of the study by Fisher’s exact test or chi-square when appropriate. Continuous variables were tested for their normality by the Kolmogorov-Smirnov test and visual inspections of QQ plots. The parametric variables were expressed as mean and standard deviation and compared between the two moments of the study by paired t-test. Non-parametric variables were expressed in median and 25 and 75% percentiles and compared between the two time points by using the Wilcoxon test.

A cluster analysis was conducted using the two-step cluster analysis algorithm with the Schwarz Bayesian criterion for clustering. The number of clusters by automatic selection and the distance were measured with log probability. The TwoStep Cluster Analysis procedure is an exploratory tool designed to reveal natural groupings within a dataset that would otherwise not be apparent. This was used in this study to group the baseline clinical variables into strongly interrelated subgroups (clusters) without prior knowledge of the target variables. We used all baseline clinical variables to build the cluster analysis that automatically resulted in two clusters. This analysis creates groups that are more similar to each other based on the log probability distances (Additional file 1). The methods of cluster analysis in the context of classifying patients in the medical field was described by McLachlan et al. [25] and this approach has been recently utilized to identify clinical variables that was related to disease phenotypes in patients with heart failure [26, 27]. A *p* value < 0.05 indicated statistical significance. All analyses were performed by using SPSS statistical software, version 20 (International Business Machines, Inc., Armonk, New York, USA). The plots were built with R (R Foundation for Statistical Computing, Vienna, Austria) version 3.4.2 with the ggplot2 package.

## Results

There were 44 patients who completed the two CMR evaluations (*n* = 88 CMR studies). The median time from transplant to the first exam was 5 days and to second exam was 189. The hematocrit was similar for the first CMR exam and preoperative value (mean difference − 0.9% [95%CI -1.437 to 3.237] *p* = 0.44). The baseline donor and recipient characteristics are detailed in Table 1 and the use of non-immunosuppressive drugs in Table 2. During the study period, no patient has developed a graft loss or a cardiovascular event (acute myocardial infarction, acute coronary syndrome, or arrhythmias). The clinical and biochemical parameters of the patients at the two time points are shown in Table 3.

**Table 1:** Donor and recipient characteristics at Baseline of study patients

	Total Patients (*n* = 44)
Age (years)	50 ± 11
Male	27 (61.4%)
Primary renal diagnosis	
Hypertension	11 (25%)
Diabetes	11 (25%)
Glomerulonephritis	8 (18.2%)
Unknown cause	7 (15.9%)
Others	7 (15.9%)
Diabetes (n. %)	14 (31.8%)
Prior Transplantation	2 (4.5%)
Length of time on hemodialysis (months)	29 [15–45]
Panel reactive antibody class I (%)	0 [0–5]
Mismatches (n)	3 [2–3]
Donor	
Deceased	37 (84.1%)
Living	7 (15.9%)
Donor Age (years)	39 ± 12
Cause of Donor Death	
Cranial trauma	15 (34%)
Cerebrovascular	15 (34%)
Others	14 (31%)
Final donor creatinin (mg/dL)	1.12 ± 0.6
Cold ischemia time (hours)	22.3 ± 6.2
Induction therapy	
Basiliximab	12 (27.3%)
Thymoglobulin	32 (72.7%)
Immunosuppression	
Tacrolimus + mycophenolate + prednisone	16 (36.4%)
	5 (11.4%)
Tacrolimus + sirolimus + prednisone	
Tacrolimus + everolimus + prednisone	23 (52.3%)

Continuous variables expressed as mean and standard deviation (mean ± standard deviation) or median and 25 and 75% percentiles (median [25th and 75th percentile]). Categorical variables expressed in number and percentage

**Table 2:** Use of non-immunosuppressive drugs at baseline and 6 months after transplantation

	Baseline (*n* = 44)	6 months (*n* = 44)	p
Aspirin	20 (45.5%)	16 (36.4%)	0.38
Calcium channel blockers	14 (31.8%)	16 (36.4%)	0.65
Beta-blockers	21 (47.7%)	27 (61.4%)	0.19
ARB/ACEi	26 (60.5%)	10 (22.7%)	0.0001
Alpha-blockers	7 (15.9%)	8 (18.2%)	0.77
Spironolactone	1 (2.3%)	1 (2.3%)	1.0
Statin	5 (11.4%)	10 (22.7%)	0.15
Ciprofibrate	0 (0%)	3 (6.8%)	0.09
Furosemide	17 (38.6%)	2 (4.5%)	0.001
Hydrochlorothiazide	3 (6.8%)	5 (11.4%)	0.45
Isosorbide	2 (4.5%)	0 (0%)	0.15

*ARB* angiotensin II receptor blockers, *ACEi* angiotensin converting enzyme inhibitor

Categorical variables expressed in number and percentage. Comparisons with chi-square test

**Table 3:** Clinical and biochemical parameters at baseline and 6 months after transplantation

	Baseline (*n* = 44)	6 months (*n* = 44)	p
HR (bmp)	86 ± 13	79 ± 12	0.013^a^
DBP (mmHg)	80 [70–90]	70 [70–80]	0.008^b^
SBP (mmHg)	135 [130–150]	120 [110–140]	0.004^b^
Body Weight (Kg)	71.1 [60.6–78]	72.6 [62–79.5]	0.17^b^
Calcium (mg/dL)	9.3 [8.9–9.6]	9.6 [9.3–10]	0.001^b^
Creatinine (mg/dL)	4.2 [2–7.7]	1.3 [1–1.8]	0.001^b^
Phosphate (mg/dL)	3.9 [3.1–5.4]	3.5 [3–3.9]	0.02^b^
Hb (g/dL)	10.7 ± 1.8	12.7 ± 1.7	0.001^a^
Ht (%)	32.7 ± 5.9	38.4 ± 4.9	0.001^a^
PTH (pg/ml)	203 [111–483]	68.8 [44.7–117]	0.001^b^

*HR* heart rate, *Hb* hemoglobin, *Ht* hematocrit, *DBP* diastolic blood pressure, *SBP* systolic blood pressure, *PTH* Parathyroid hormone

Continuous variables expressed as mean and standard deviation (mean ± standard deviation) or median and 25 and 75% percentiles (median [25th and 75th percentile])

^a^Paired t test; ^b^Wilcoxon test

### LV mass and function

There was a trend to improved LV function after 6 months, *p* = 0.07 but no statistical difference in LV mass index (Table 4).

**Table 4:** Left ventricular parameters of cardiac magnetic resonance at baseline and 6 months after transplantation

	Baseline (*n* = 44)	6 months (*n* = 44)	p
Ejection Fraction (%)	64 ± 12	67 ± 10	0.07^a^
EDV (ml)	155 ± 43	152 ± 35	0.61^a^
ESV (ml)	52 [38–69]	50 [39–63]	0.49^b^
SV (ml)	96 ± 21	100 ± 20	0.27^a^
LVM (g)	154 ± 40	152 ± 35	0.76^a^
EDVi (mL/m^2^)	88 ± 23	85 ± 19	0.29^a^
ESVi (mL/m^2^)	30 [21–40]	28 [22–36]	0.28^b^
SVi (mL/m^2^)	55 ± 12	56 ± 11	0.42^a^
LVMi (g/m^2^)	87 ± 20	85 ± 16	0.36^a^
IS (mm)	12.9 ± 3.3	12.7 ± 3.2	0.71^a^
LVPW (mm)	10.2 ± 2.2	9.6 ± 1.8	0.07^a^
LVED (mm)	53 ± 7	52 ± 7	0.21^a^
LVES (mm)	34 ± 9	33 ± 6	0.20^a^

*BSA* body surface area, *EDV* end-diastolic volume, *EDVi* end-diastolic volume indexed to BSA, *ESV* end-systolic volume, *ESVi*, end-systolic volume index to BSA, *LVM* left ventricular mass, *LVMi* left ventricular mass indexed to BSA, *SV* systolic volume, *SVi* systolic volume index to BSA, *IS* interventricular septum, *LVPW* left ventricular posterior wall, *LVED* left ventricular end-diastolic diameter, *LVES* left ventricular end-systolic diameter

Continuous variables expressed as mean and standard deviation (mean ± standard deviation) or median and 25 and 75% percentiles (median [25th and 75th percentile])

^a^paired t-test; ^b^Wilcoxon test

### Native T1 times (primary outcome)

**Fig. 2:**
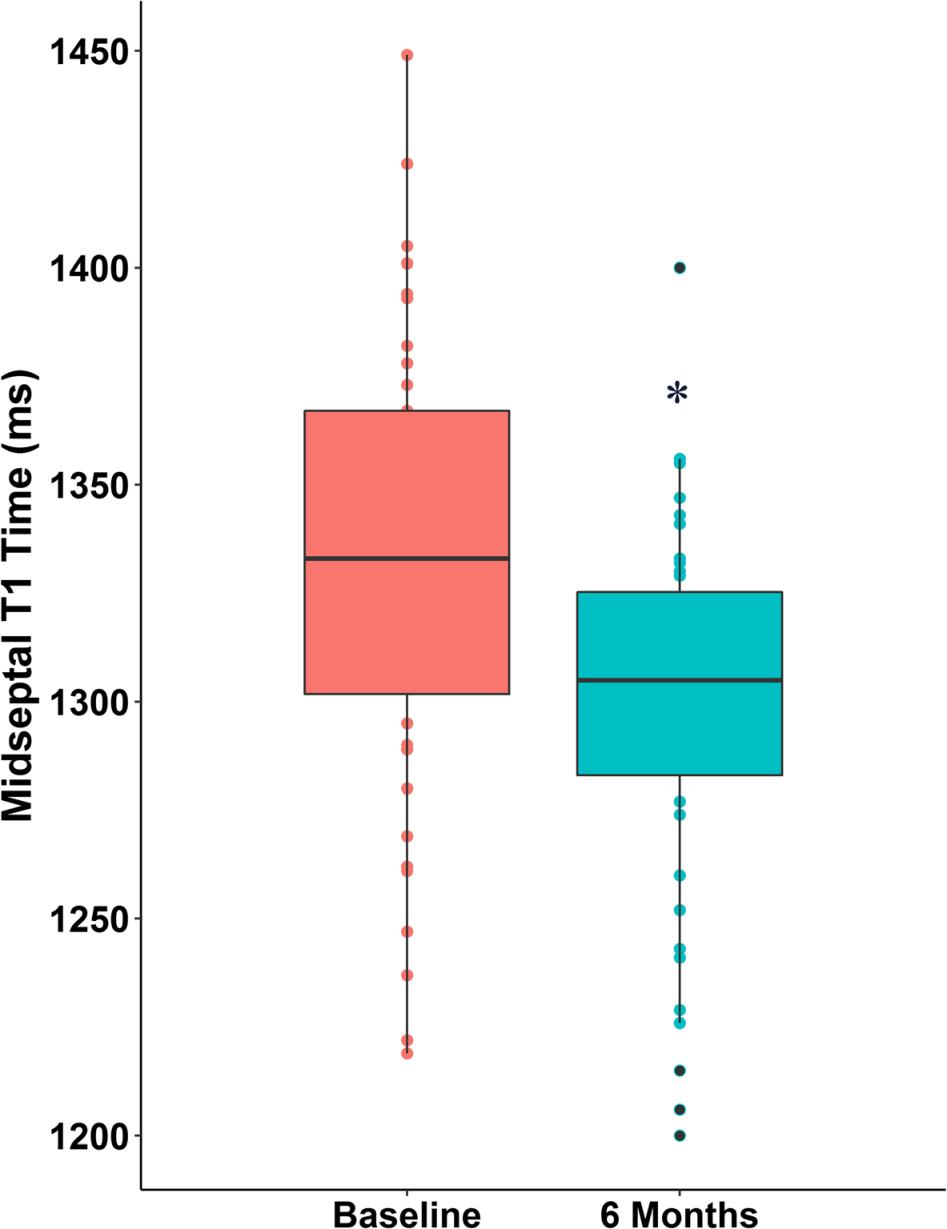
Boxplot comparing midventricular septum T1 time at baseline and 6 months after transplantation.* *p* < 0.001 x baseline

**Fig. 3:**
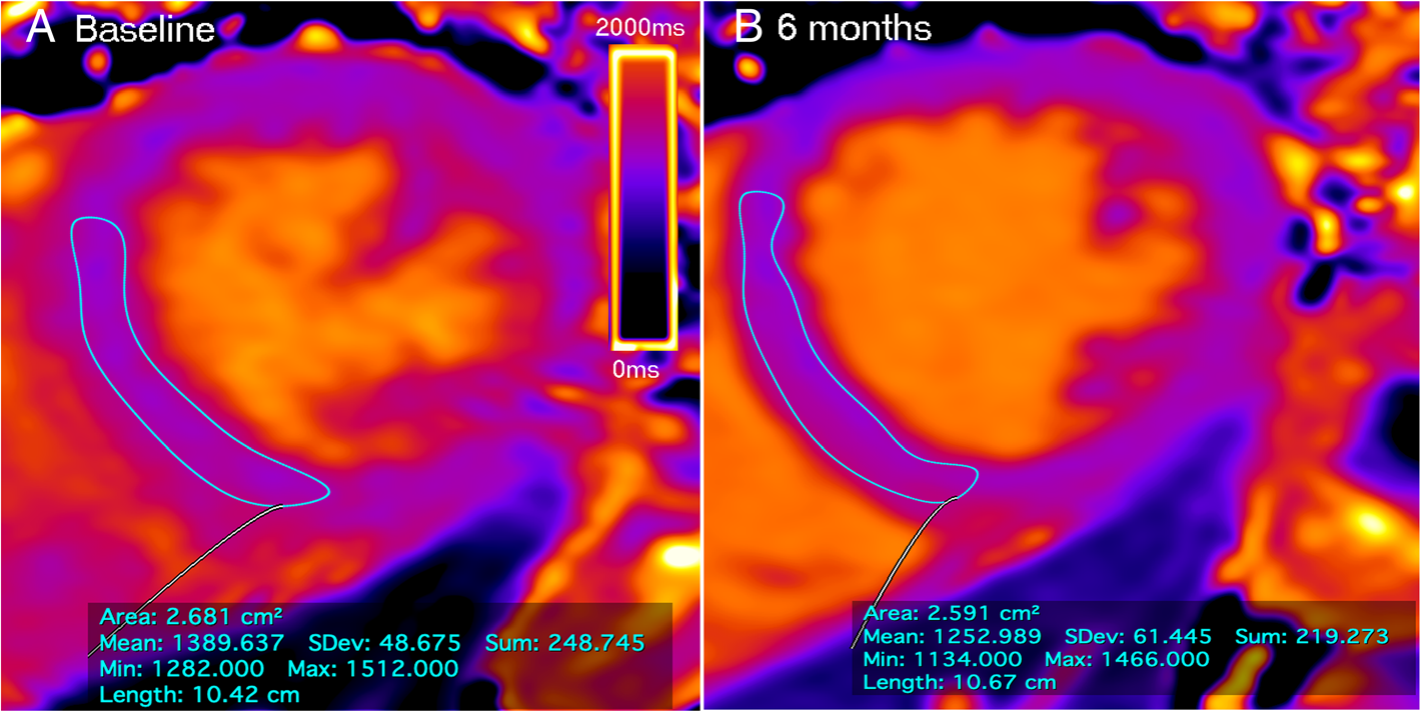
Example of native T1 mapping in a 38-year-old deceased-donor kidney transplant patient. The T1 value changing from 1389 ms (**a**) to 1252 ms (**b**) showing a decrease 6 months after transplantation (visual pattern with increased blue staining, patient id = 3).The thin line denotes the manually contoured region of interest (ROI) in the midventricular septum. The box shows the measures of T1 value by automatic computer average of the pixels. The color map scale represents the values in milliseconds (blue corresponds to smaller values and yellow to higher values). Data acquired with the modified look-locker inversion recovery (MOLLI) T1 mapping sequence at field strength of 3 T

The data analysis revealed a significant reduction in native T1 time 6 months after renal transplantation. The mean T1 decreased from 1331 ± 52 ms to 1298 ± 42 ms at 6 months (*p* < 0.001) (Fig. 2). The analysis of individual cases of native T1 demonstrated that the majority of the patients reached a reduction in T1 values between baseline and 6 months after transplantation (Additional file 2). Figure 3 display an example of the T1 map at the two time periods in three patients: one patient with native T1 maintenance, one with an increase, and another with a decrease in native T1 times.

### Cluster analysis

The 44 patients were divided into 2 groups by two-step cluster algorithm. In cluster-1 (*n* = 30) there were no patients with diabetes mellitus and in cluster-2 (*n* = 14), all 14 patients (100%) had diabetes (*p* < 0.001, Table 5). The LV mass index at baseline was significantly higher in cluster-2 (93 ± 19 g/m^2^) than in cluster-1 (83 ± 17 g/m^2^, *p* = 0.02).

**Table 5:** Demographic and clinical characteristics of patients divided in to clusters by Two Step Algorithm

	Cluster-1 (*n* = 30)	Cluster-2 (*n* = 14)	p
Age (years)	48 ± 11	54 ± 08	0.10
Male	20 (66.7%)	7 (50%)	0.29
Primary renal diagnosis			
Hypertension	8 (26.7%)	3 (21.4%)	
Diabetes	0 (0%)	11 (78.6%)	
Glomerulonephritis	8 (26.7%)	0 (0%)	0.001
Unknown cause	7 (23.3%)	0 (0%)	
Others	7 (23.3%)	0 (0%)	
Diabetes (n. %)	0 (0%)	14 (100%)	0.0001
Prior Transplant	2 (6.7%)	0 (0%)	0.32
Length of time on hemodialysis (months)	28 [14–43]	33 [17–47]	0.51
Panel reactive antibody class I (%)	0 [0–15]	0 [0–0]	0.33
Mismatches (n)	3 [2–4]	3 [2–3]	0.20
Donor			
Deceased	24 (80%)	13 (92.9%)	0.27
Living	6 (20%)	1 (7.1%)	
Donor Age (years)	40 ± 10	37 ± 13	0.40
Cause of Donor Death			
Cranial trauma	9 (30%)	6 (42%)	
Cerebrovascular	9 (30%)	4 (28.5%)	
Others	7 (40%)	4 (28.5%)	0.90
Final donor creatinin (mg/dL)	1.19 ± 0.6	1.14 ± 0.58	0.78
Cold ischemia time (hours)	21.8 ± 7	23.2 ± 3.9	0.50
Induction therapy			
Basiliximab	9 (30%)	3 (21.4%)	0.55
Thymoglobulin	21 (70%)	11 (78.6%)	
Immunosuppression			
FK + MFS + PDN	13 (43.3%)	3 (21.4%)	
FK + SRL + PDN	4 (13%)	1 (7.1%)	0.22
FK + EVR + PDN	13 (43.3%)	10 (71.4%)	
Use of ARB/ACEi	17 (56.7%)	9 (69.2%)	0.43
Calcium (mg/dL)	9.39 ± 0.80	9.04 ± 0.52	0.14
Phosphate (mg/dL)	4.19 ± 1.78	4.5 ± 1.4	0.47
PTH (ng/dl)	199 [117–499]	211 [68–470]	0.76
Hb (g/dl)	10.8 ± 1.9	10.6 ± 1.7	0.72
SBP (mmHg)	138 ± 25	139 ± 13	0.96
DBP (mmHg)	85 ± 14	77 ± 12	0.07
LVMi (g/m^2^)	83 ± 17	93 ± 19	0.02

*FK + EVR + PDN* Tacrolimus, everolimus and prednisone, *FK + MFS + PDN* tacrolimus, mycophenolate and prednisone, *FK + SRL + PDN* tacrolimus, sirolimus and prednisone, *Hb* hemoglobin, *LVMi* left ventricular mass index, *DBP* diastolic blood pressure, *SBP* systolic blood pressure, *PTH* Parathyroid hormone, *ARB* angiotensin II receptor blockers, *ACEi* angiotensin converting enzyme inhibitor

Continuous variables expressed as mean and standard deviation (mean ± standard deviation) or median and 25 and 75% percentiles (median [25th and 75th percentile]). Categorical variables expressed in number and percentage

The analysis of LV mass demonstrated that in cluster-1 the LV mass index decreased from 84.5 ± 19.7 g/m^2^ (baseline) to 81.5 ± 14.1 g/m^2^ (6 months; *p* = 0.32). In cluster-2, the LV mass index did not change (93 ± 21.1 g/m^2^ (baseline) to 92.2 ± 17.2 g/m^2^ (6 months; *p* = 0.86). (Additional file 3).

The LV ejection fraction (EF) changed from 64.1 ± 13.5% (baseline) to 66.3 ± 8.5% (6 months) in cluster-1, *p* = 0.24. In cluster-2, the EF changed from 65.3 ± 9.8% (baseline) to 68.6 ± 12.2% (6 months), *p* = 0.09.

**Fig. 4:**
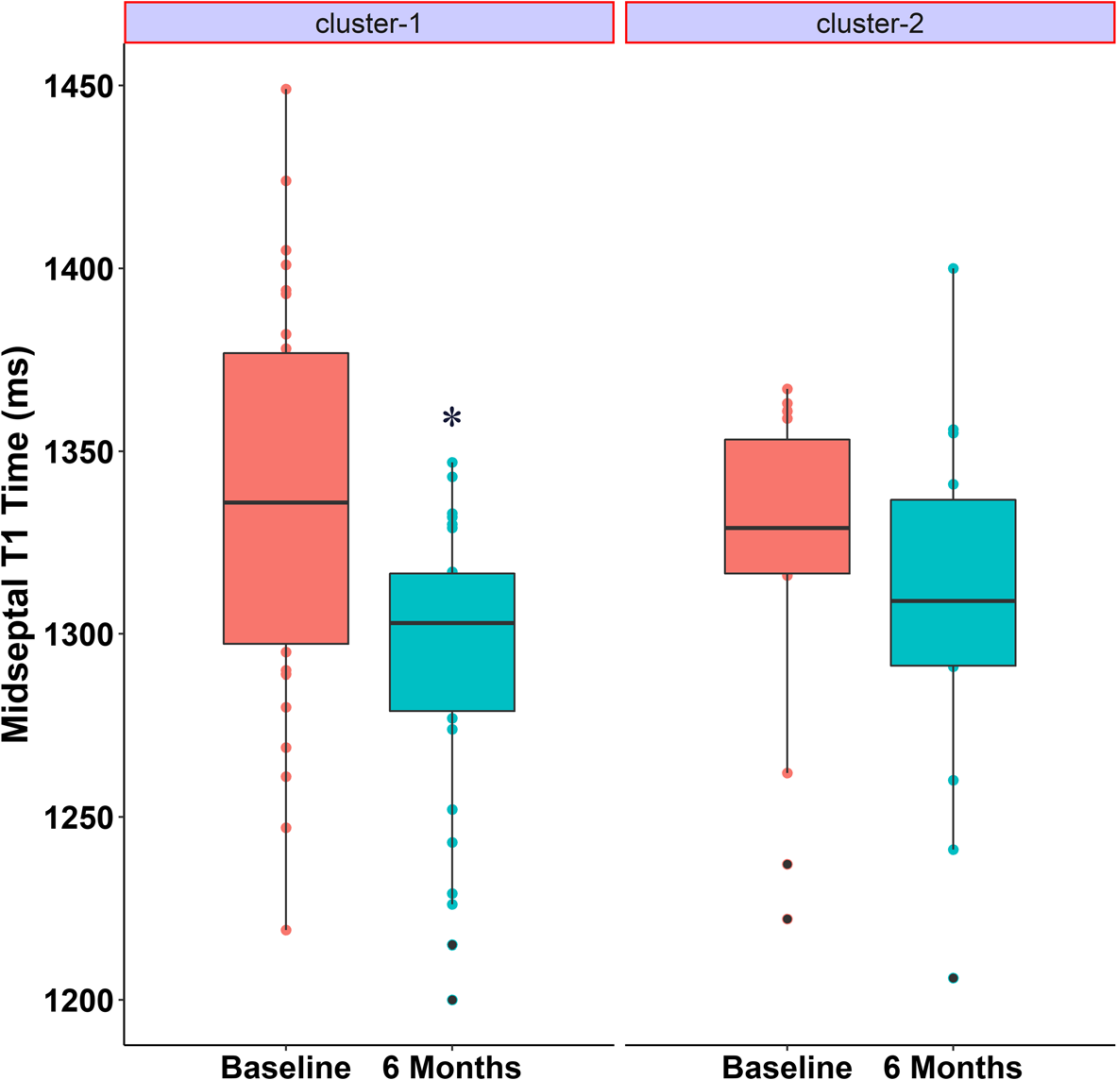
Boxplot comparing midventricular septum T1 time at baseline and 6 months after transplantation splitted into two groups. The groups were wrapped by a cluster analysis designed to reveal natural groupings within a dataset that would otherwise not be apparent. The majority of patients in cluster-2 were diabetics and the patients with greater baseline left ventricular mass index. * *p* = 0.001 x baseline

In cluster-1, native T1 values decreased from 1337 ± 54.8 ms (baseline) to 1293 ± 38.7 ms (6 months; *p* = 0.001). In cluster-2, the native T1 was similar at baseline and at 6 months (*p* = 0.50, Fig. 4). The analysis of individual cases demonstrates that only the patients in Cluster-1 reached a reduction in T1 values between baseline and 6 months after transplantation (Additional file 4).

## Discussion

To our knowledge, this is the first prospective study to evaluate the change in myocardial native T1 after renal transplantation. We demonstrated that native T1 significantly decreased 6 months after transplantation.

### Use of non-immunosuppressive drugs

The inhibition of the renin-angiotensin-aldosterone system, induced by angiotensin-converting enzyme inhibitors (ACEIs) and angiotensin II receptor blockers (ARBs), reduces myocardial fibrosis, regardless of its hypotensive action [28]. In addition, the selective AT1 angiotensin II antagonist prevents myocyte hypertrophy and interstitial fibrosis [29]. Most patients use ARBs or ACEIs during the dialysis period. In the early postoperative period after transplantation, however, these medications are frequently discontinued since they impair renal graft function, perpetuate anemia, and leads to hyperkalemia. In the present study, 6 months after transplantation, the use of ARBs/ACEIs decreased significantly; thus, the use of these medications was not the main factor to the improvement in native T1 after transplantation. Additionally there was no correlation between using ACEIs/ARBs and change in native T1 (data not shown).

### Clinical and biochemical parameters

We found that at 6 months after renal transplantation, heart rate and systolic and diastolic blood pressures decreased significantly. This finding probably resulted from inactivation of the sympathetic system and the renin-angiotensin-aldosterone system after transplantation. Both systems, when activated, stimulate intracellular signaling pathways, with consequent increase in protein synthesis in myocytes and fibroblasts leading to cellular hypertrophy and fibrosis [30]. Other effects are the activation of growth factors, activation of metalloproteinases, hemodynamic overload by vasoconstriction and water retention, increase in oxidative stress, and direct cytotoxic effect, leading to cell death due to necrosis or apoptosis [31–33]. Blockade of these systems plays an important role in preventing or attenuating the developmental process for replacement fibrosis.

### CMR

In the CMR evaluation, considering ventricular function, volume and mass, we did not find differences at 6 months, similar to Patel [34] and Prasad et al. [35]. Patel performed the first study to serially evaluate the LVs of patients who have undergone kidney transplantation using CMR. Prasad results showed a result deriving from an adjustment of LV parameters using a gender-specific method.

In contrast, improved CMR derived LV parameters have been demonstrated in children within 6 months after transplantation [36], indicating that gender adjustment assumes particular importance in adults. The pediatric patients also had a short time on dialysis (median = 6 months), perhaps explaining lower abnormalities in cardiac structure and function. Additionally, different CMR technology, distinct reference populations and diverse study design might be the reason for this discrepancy.

In a different way from the ventricular function and mass, the native T1 was significantly reduced 6 months after renal transplantation. The native T1 time is a sensitive marker of myocardial fibrosis [19, 37–39] and we associated the decrease of native T1 with the reduction of reactive fibrosis after renal transplantation. Reactive fibrosis is secondary to neurohumoral activation that was triggered by the cellular mediators angiotensin II, aldosterone and endothelin I, [40–42] which stimulate the myofibroblasts to increase the production and deposition of collagen in the ECM. At this time, no cardiomyocyte necrosis is detected and the fibrosis can be discontinued. Thus, renal transplantation has the potential to inhibit this neurohumoral activation by improving volume overload and pressure control and thereby contribute to the regression of reactive fibrosis and prevent the progression to irreversible replacement fibrosis.

To date, no prior studies have evaluated native T1 in renal transplant recipients. Using 3 T CMR and measuring T1 times in the midventricular septum, Rutherford and Graham-Brown et al. found lower native T1 values in hemodialysis patients than the baseline values of the present study [20, 38]. An explanation for these differences may be that in the present study the mean dialysis time was longer (38 months) than that in Rutherford and Grahan-Brown’s studies (5 and 21 months, respectively) [20, 38]. There is still no standardization of native T1 values for the normal population that can differentiate between disease states and normality [43, 44], although Weingärtner et al., using a protocol similar to that of the present study (MOLLI with 3 T CMR) in 20 healthy subjects, found a native T1 of 1183 ± 47 ms [44]. We also observed that T1 values after 6 months of transplantation was not similar to normal healthy population. One explanation is that it would take a longer time to achieve values similar to those in a healthy population. Finally, it is likely that transplantation may not be able to fully reverse the myocardial fibrosis.

The discrepancies between ventricular mass/function and fibrosis in the present study are related to the specificity of the method. Thus, the studies that demonstrated a reduction of ventricular mass in post-transplant were performed using transthoracic echocardiography, which may be influenced by volume overload [45]. Furthermore, echocardiography assumes an approximately cubic LV shape when calculating the LV mass index [46]. On the other hand, the myocardial fibrosis in patients with advanced CKD [4] is better accessed by CMR, using the native T1 measurement [38, 39]. Thus, native T1 may be better correlated to morphological abnormalities in the myocardium of chronic renal patients [11, 20].

In order to explore hidden patterns in the data, we performed a cluster analysis, and a group showed no improvement of native T1. The main finding of this group was the greater rate of diabetes. Previous studies have demonstrated that high glucose concentrations can lead to pathological changes in the myocardium, including the accumulation of ECM proteins. Mechanisms responsible for these alterations may include overproduction, decreased degradation, and / or chemical modifications of ECM proteins [47]. Consequently, in diabetic patients the transplantation cannot interrupt these mechanisms that can explain the lack of decrease in native T1. In addition to diabetes, another characteristic of this cluster group is the greater baseline LV mass index. Patients with higher LV mass index also did not reach a decrease in T1 and myocardial fibrosis, possibly because these patients achieved such a striking change in cardiac structure that it became unlikely to be reversed.

### Study limitations

Although native T1 time has demonstrated a positive correlation with biopsy-proven fibrosis in diseases with pressure overload [21], we could not perform histological confirmation in this study. Thus, in the absence of confirmatory biopsy, a direct inference between native T1 and myocardial fibrosis is not allowed. Another limitation was the baseline CMR at the time of transplantation. Although performing the pre-transplant CMR would be more appropriate, this is not possible with deceased donors due to long waiting list times. Then, we performed the first CMR as close to the transplant surgery as possible. Native T1 is more stable than blood T1 for variations in hematocrit and heart rate [48]. Furthermore, we conduced additional analyzes controlled for these factors and the results of native T1 were not affected by these confounders (Additional file 5). Although of sensitivity analysis, the decrease of hematocrit in the postoperative period may interfere with the analysis of native T1 resulting in lower T1 values. Additional studies with longer observation time are needed to evaluate the sequential changes of native T1 and parameters of left ventricular mass and function. However, this work is the first to evaluate the myocardium of renal transplant patients using native T1 time. The images were performed in the same apparatus, with the same magnetic field (3 T), and all the patients underwent the same imaging protocol and analyzed by a single blinded observer.

In conclusion, myocardial native T1 decreased significantly 6 months after renal transplantation. Patients that do not achieve a significant decrease in native T1 include those with diabetes and those with highest baseline LV mass index. The measurement of native T1 time by CMR may be considered an appropriate complementary method to evaluate myocardial fibrosis in renal transplant patients without using contrast.

## Additional files

Additional file 1: Statistical analysis of Two-Step Cluster. Cluster sizes and importance of the predictors. (DOCX 38 kb)

Additional file 2: Analysis of individual cases of native T1 map at baseline and 6 months after transplantation (*n* = 44 before and *n* = 44, 6 months after transplantation). (TIFF 439 kb)

Additional file 3: A Boxplot comparing left ventricular mass index (LVMi) at baseline and 6 months after transplantation. B: Analysis of individual cases of LVMi at baseline and 6 months after transplantation. C: Boxplot comparing Left Ventricular Mass index (LVMi) at baseline and 6 months after transplantation split into two groups (clusters). D: Analysis of individual cases of native T1 map at baseline and 6 months after transplantation splitted into two groups by Cluster analysis. (TIF 1406 kb)

Additional file 4: Analysis of individual cases of native T1 map at baseline and 6 months after transplantation splitted into two groups by Cluster analysis. (*n* = 44 before and *n* = 44, 6 months after transplantation). (TIFF 268 kb)

Additional file 5: Additional statistical analyzes for native T1 adjusted for confounding factors. (DOCX 20 kb)

## Abbreviations

ACEi: Angiotensin-converting enzyme inhibitor
ARB: Angiotensin II receptor blockers
CKD: Chronic kidney disease
CMR: Cardiovascular magnetic resonance
CVD: Cardiovascular disease
DM: Diabetes mellitus
ECM: Extracellular matrix
ECV: Extracellular volume fraction
EF: Ejection fraction
FOV: Field of view
HD: Hemodialysis
LV: Left ventricle/left ventricular
MOLLI: Modified look-locker inversion recovery
ROI: Region of interest
