# Supplementary material for: Kidney transplantation is associated with reduced myocardial fibrosis. A cardiovascular magnetic resonance study with native T1 mapping
Source: J Cardiovasc Magn Reson. 2019 Mar 27;21:21. doi: 10.1186/s12968-019-0531-x (PMC6437926; doi:10.1186/s12968-019-0531-x)
Supplement: Supplementary file 1 — Statistical analysis of Two-Step Cluster. Cluster sizes and importance of the predictors. (DOCX 38 kb) [file 12968_2019_531_MOESM1_ESM.docx]

The Two-Step Cluster analysis results in two clusters that better separate the groups without prior knowledge of the target variables (figure 1). The baseline variables used to create the cluster and the importance of predictors was described in figure 02. To measure the distance we used the **Log-likelihood.** The likelihood measure places a probability distribution on the variables. Continuous variables are assumed to be normally distributed, while categorical variables are assumed to be multinomial. All variables are assumed to be independent. The number of clusters was d**etermined automatically.** The procedure will automatically determine the "best" number of clusters, using the Schwarz Bayesian criterion. These analyses were conducted using SPSS version 20.

**Figure 01. Two-Step Cluster Sizes.**

**Figure 02. Baseline variables and importance of predictors in Two-Cluster algorithm.**
