# Supplementary figures and images for: Kidney transplantation is associated with reduced myocardial fibrosis. A cardiovascular magnetic resonance study with native T1 mapping
Source: J Cardiovasc Magn Reson. 2019 Mar 27;21:21. doi: 10.1186/s12968-019-0531-x (PMC6437926; doi:10.1186/s12968-019-0531-x)

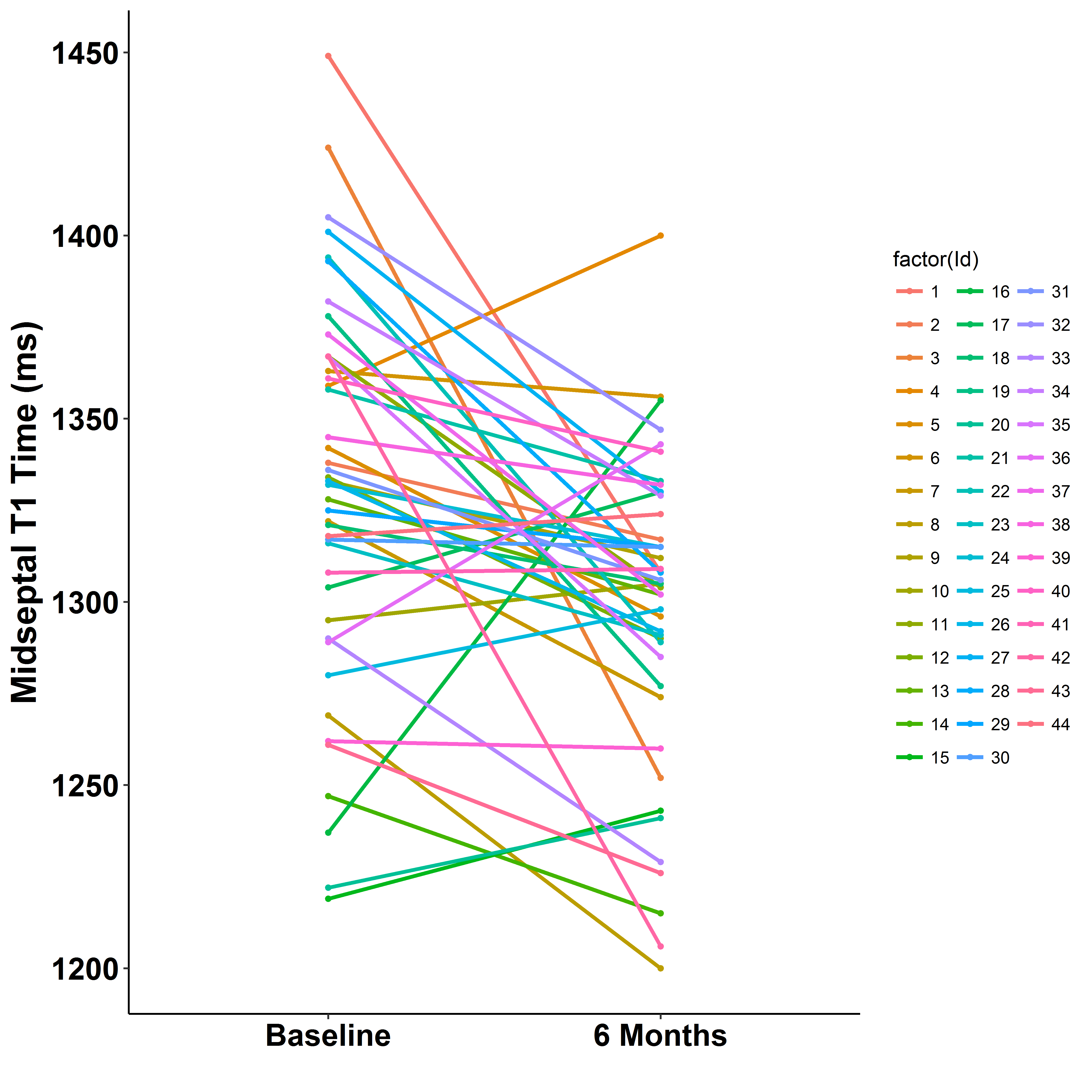

Supplement: Supplementary file 2 — Analysis of individual cases of native T1 map at baseline and 6 months after transplantation (n = 44 before and n = 44, 6 months after transplantation). (TIFF 439 kb) [file 12968_2019_531_MOESM2_ESM.tiff]

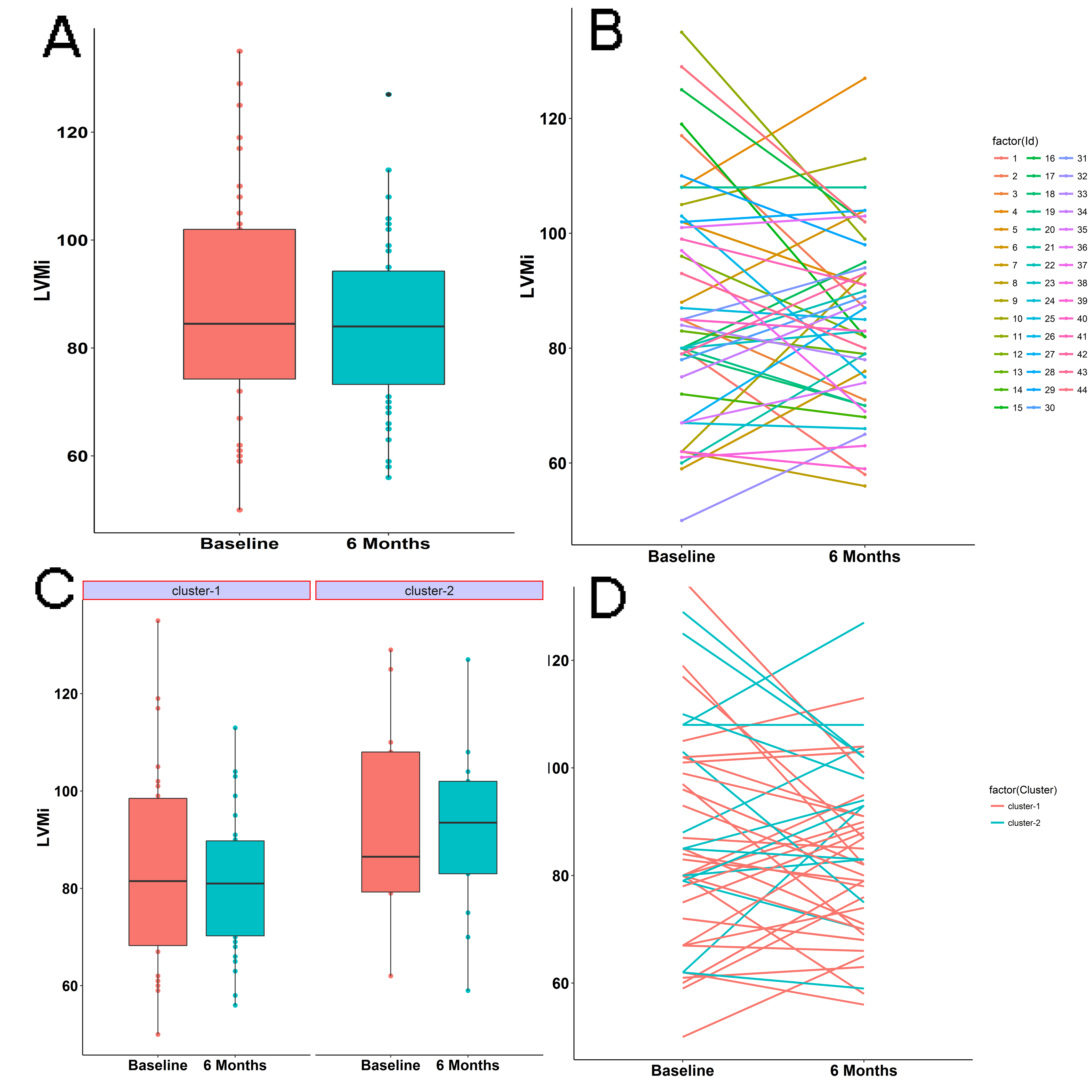

Supplement: Supplementary file 3 — A Boxplot comparing left ventricular mass index (LVMi) at baseline and 6 months after transplantation. B: Analysis of individual cases of LVMi at baseline and 6 months after transplantation. C: Boxplot comparing Left Ventricular Mass index (LVMi) at baseline and 6 months after transplantation split into two groups (clusters). D: Analysis of individual cases of native T1 map at baseline and 6 months after transplantation splitted into two groups by Cluster analysis. (TIF 1406 kb) [file 12968_2019_531_MOESM3_ESM.tif]

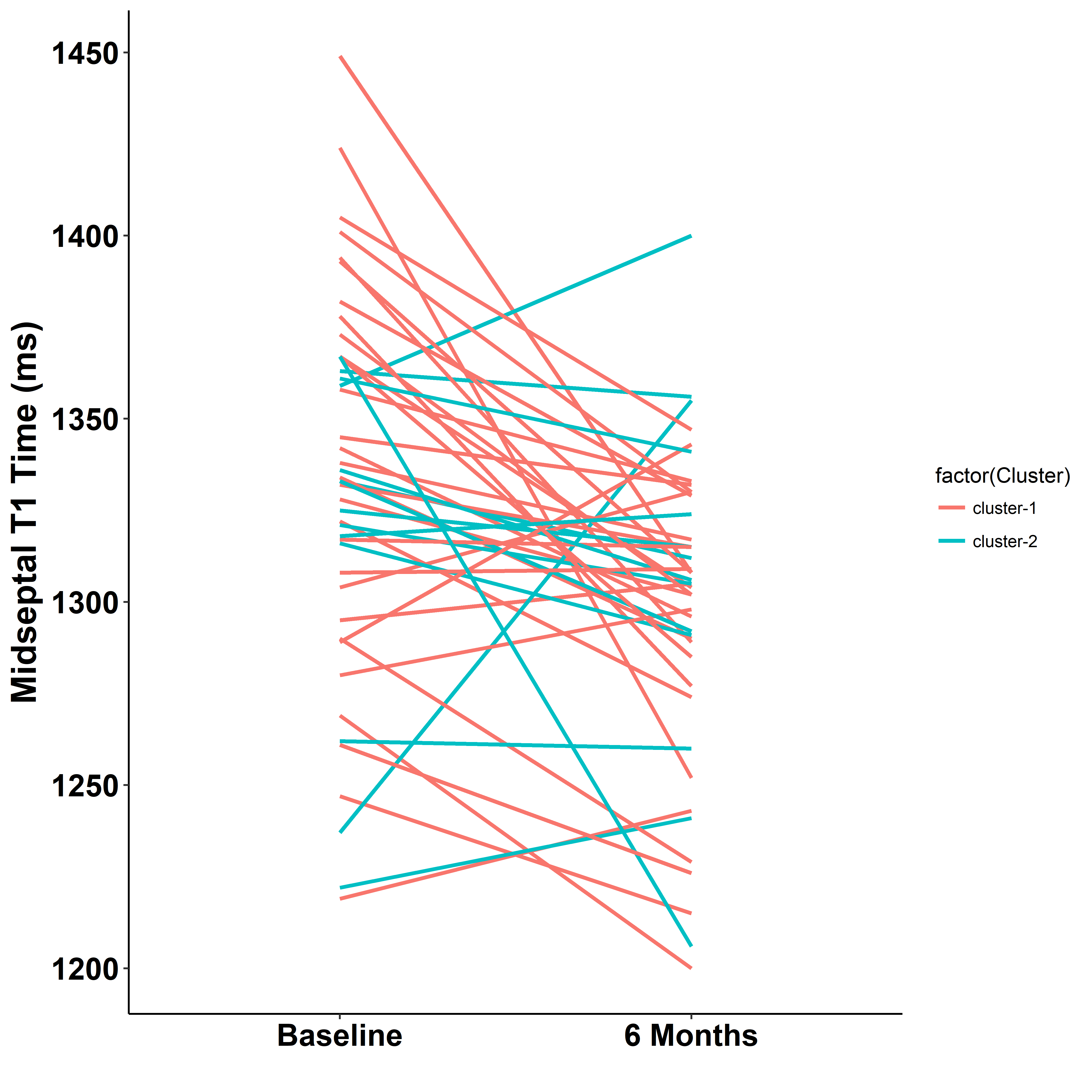

Supplement: Supplementary file 4 — Analysis of individual cases of native T1 map at baseline and 6 months after transplantation splitted into two groups by Cluster analysis. (n = 44 before and n = 44, 6 months after transplantation). (TIFF 268 kb) [file 12968_2019_531_MOESM4_ESM.tiff]
