# Supplementary material for: Kidney transplantation is associated with reduced myocardial fibrosis. A cardiovascular magnetic resonance study with native T1 mapping
Source: J Cardiovasc Magn Reson. 2019 Mar 27;21:21. doi: 10.1186/s12968-019-0531-x (PMC6437926; doi:10.1186/s12968-019-0531-x)
Supplement: Supplementary file 5 — Additional statistical analyzes for native T1 adjusted for confounding factors. (DOCX 20 kb) [file 12968_2019_531_MOESM5_ESM.docx]

**Addiction statistical analysis of native T1 adjusted for confounders**

The median time to the first exam was 5 days. The pre-operatory mean hematocrit was 33.6±5.1 and at the time of examination of 32.7±5.9 (without statistical differences, p=0.44). There was an increase in hematocrit and hemoglobin at 6 months, which is an expected effect of transplantation due to the improvement of renal function. The native T1 may be affected by hematocrit and heart rate. To prove that these confounders did not affect the native T1 time, we conducted a linear mixed model of repeated measures. This analysis considers the repeated measures and the covariance structure. For the repeated measurements, the first-order autoregressive structure was used as covariance structure. As fixed effects we used the time of the evaluation (baseline and 6 months), heart rate, hematocrit and body weight. As random factor we used patients to better assess individual variations. We perform additional models including other confounding variables at model 2 and 3. These Statistical analyses were performed using SPSS software, version 20 (IBM, Armonk, NY).

**Model 01. Linear mixed models to native T1 adjusted to confounders (hematocrit, heart rate and weight) over time (baseline and 6 months)**

| **Model Term** | | **Coefficient p** | |
| --- | --- | --- | --- |
| **Intercept** |  | 1,295.2 | 0.000 |
| **Hematocrit (%)** |  | -0.707 | 0.432 |
| **Heart Rate** |  | 0.334 | 0.422 |
| **Weight (kg)** |  | 0.046 | 0.600 |
| **Time (6-months)** |  | 26.52 | 0.007 |
| **Time (baseline)** |  | 0^a^ |  |
|  |  |  |  |

Probability distribution: Normal

Link function: Identity

a. This coefficient is set zero because it is redundant

**Model 02. Linear mixed models to native T1 adjusted to confounders (hematocrit, heart rate, weight and Left ventricular mass index at baseline) over time (baseline and 6 months)**

| **Model Term** | | **Coefficient p** | |
| --- | --- | --- | --- |
| **Intercept** |  | 1,292.2 | 0.000 |
| **Hematocrit (%)** |  | -0.709 | 0.435 |
| **Heart Rate** |  | 0.335 | 0.422 |
| **Weight (kg)** |  | 0.047 | 0.597 |
| **LVMi (g/m^2^)** |  | 0.034 | 0.912 |
| **Time (6-months)** |  | 26.38 | 0.008 |
| **Time (baseline)** |  | 0^a^ |  |
|  |  |  |  |

LVMi: left ventricular mass index

Probability distribution: Normal

Link function: Identity

a. This coefficient is set zero because it is redundant

**Model 03. Linear mixed models to native T1 adjusted for confounders (hematocrit, heart rate, weight, left ventricular mass index at baseline, diabetes and use of ARB/ACEs over time (baseline and 6 months)**

| **Model Term** | | **Coefficient p** | |
| --- | --- | --- | --- |
| **Intercept** |  | 1,283.6 | 0.000 |
| **Hematocrit (%)** |  | -0.667 | 0.491 |
| **Heart Rate** |  | 0.375 | 0.398 |
| **Weight (kg)** |  | 0.047 | 0.607 |
| **LVMi (g/m^2^)** |  | 0.034 | 0.912 |
| **Diabetes** |  | 4.259 | 0.763 |
| **Use of ARB/ACEs** |  | -1.200 | 0.922 |
| **Time (6-months)** |  | 25.42 | 0.017 |
| **Time (baseline)** |  | 0^a^ |  |
|  |  |  |  |

LVMi: left ventricular mass index

ARB/ACEs: angiotensin II receptor blockers; angiotensin converting enzyme inhibitor

Probability distribution: Normal

Link function: Identity

a. This coefficient is set zero because it is redundant
